# Supplementary material for: Neural representation of visual concepts in people born blind
Source: Nat Commun. 2018 Dec 7;9:5250. doi: 10.1038/s41467-018-07574-3 (PMC6286313; doi:10.1038/s41467-018-07574-3)
Supplement: Supplementary file 1 — Supplementary Information [file 41467_2018_7574_MOESM1_ESM.pdf]

Neural representation of visual concepts in people born blind

Striem-Amit, Wang et al.

Supplementary Material

Supplementary Notes

In addition to the adATL cluster, an additional distinct patch more posterior in location on STS, pdATL, also showed the imperceptibility X group interaction (**Fig. 2A**). However, this cortical area does not seem to be associated with a significant imperceptibility effect in the blind group, in either the univariate or multivariate analyses (**Fig. 2C, Fig. 3B**). To clarify this region's role, we further investigated its responses to perceptible and imperceptible concepts in the blind. Sampling the cluster pdATL (defined from Experiment 1 imperceptibility X group interaction) in Experiment 2, we find that this region does not show a main imperceptibility effect ($F(1,11)=1.7$, $p<0.21$) in the blind, but rather inconsistent responses for different conceptual domains (**Supplementary Fig. 6A**). The imperceptibility X group interaction arises from a bias for imperceptible items in the sighted group (**Supplementary Fig. 6B**). Furthermore, pdATL shows a domain X imperceptibility interaction at the ROI level in the blind, suggesting imperceptibility is not itself a determining factor in this region ($F(2,22)= 5.73$, $p< 0.01$; for whole brain interaction analysis see **Supplementary Fig. 6E**). Therefore, the functional properties of the pdATL area are not clear based on our data and should be elucidated in future work.

Supplementary Figures

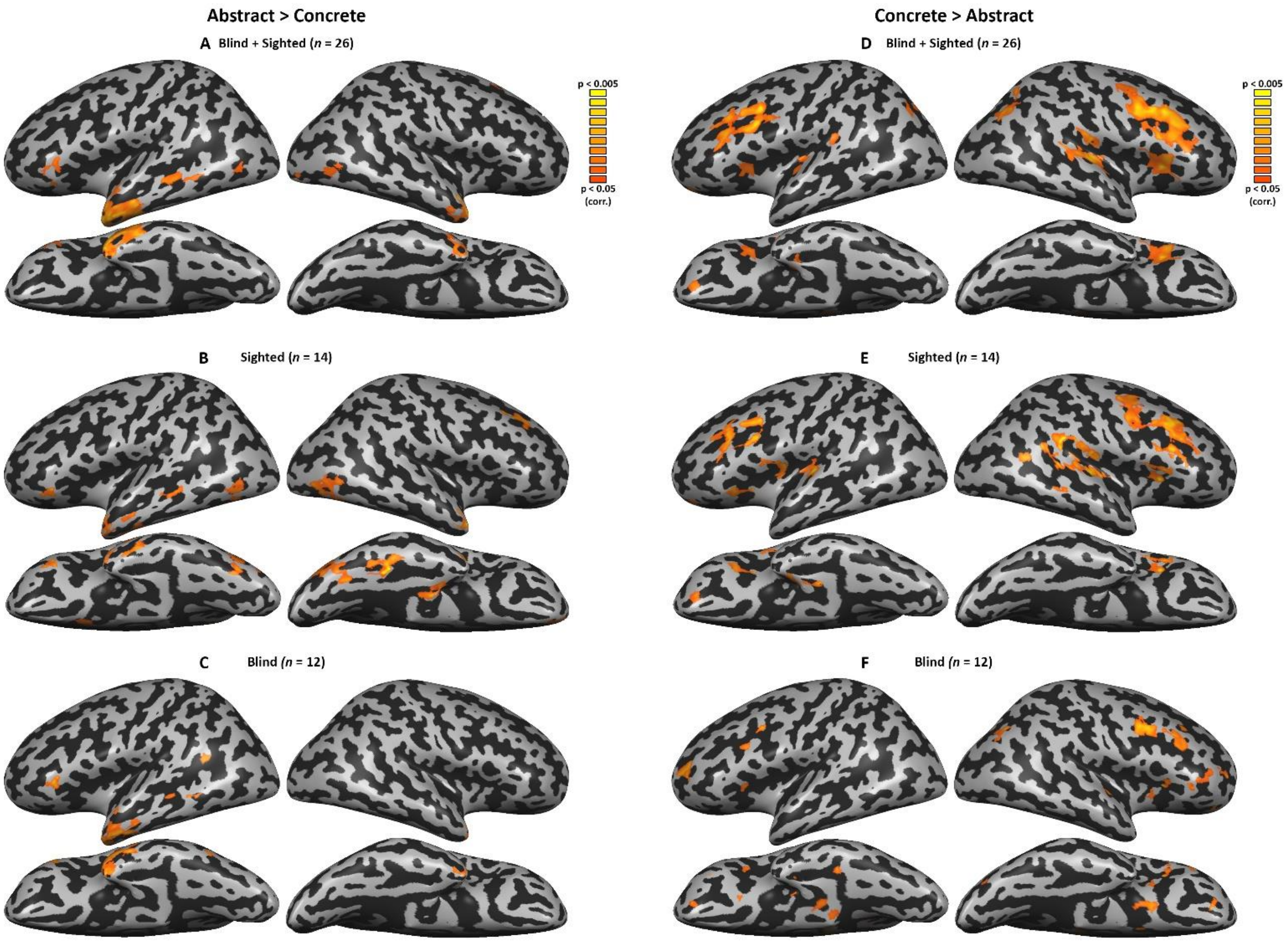

Supplementary Figure 1: Main effects for abstract and concrete concepts in each group

The whole-brain, random effect contrasts of abstract > concrete concepts (**left panels**) and concrete > abstract (**right panel**) are depicted for the entire group of subjects (n=26, **A,D**) sighted subjects only (n=14, **B, E**) and blind subject group (n=12, **C, F**). Data from Exp. 1.

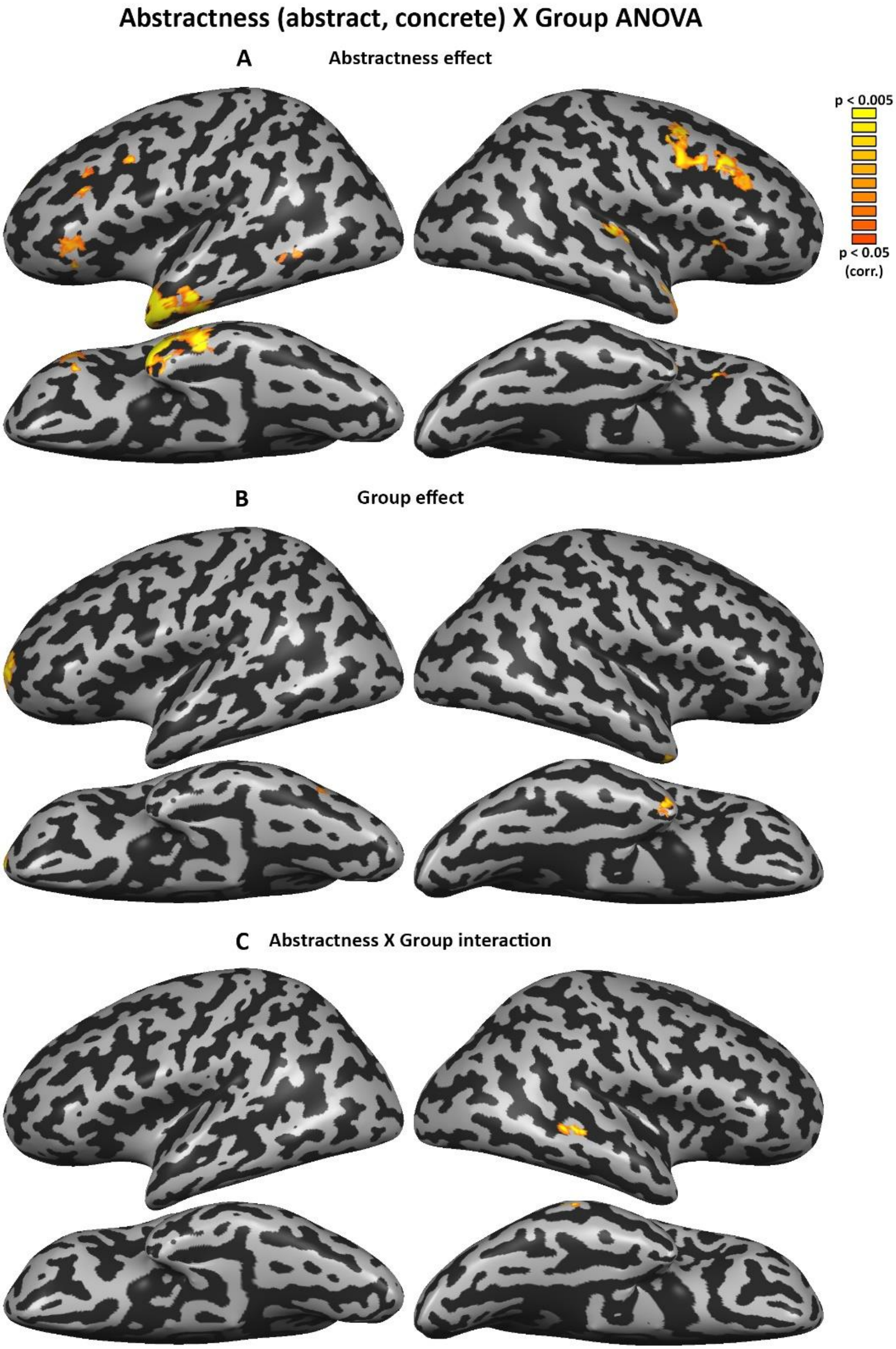

Supplementary Figure 2: Group effects in the comparison between abstract and concrete concepts

A 2-way ANOVA was computed for Abstractness (**A**; abstract vs. concrete concepts) and Group effects (**B**; blind, sighted; data from Exp.1). While the abstractness effect is found in left ATL across groups, neither group effect or abstractness X group interaction (**C**) is found in this region.

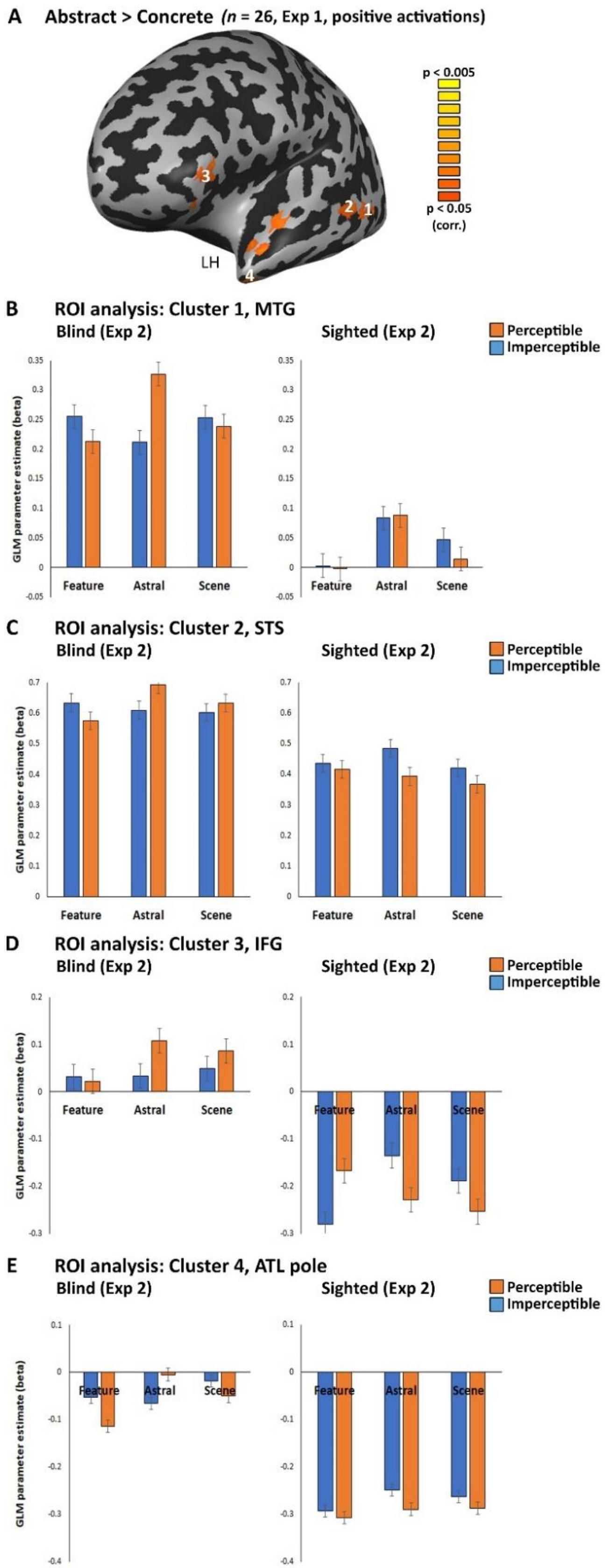

Supplementary Figure 3: No group X imperceptibility interaction in the abstract concept network outside ATL

A. Replica of Fig. 1C: A contrast of typical abstract words (e.g. "freedom") with concrete everyday objects (e.g. "cup") in conjunction with abstract words significant activation in the combined subject group (n=26; data

from Exp. 1). Clusters outside dorsolateral ATL are numbered, and data was sampled from Exp. 2 from each of these, none showing significant group X imperceptibility interaction.

B. Data sampled from Cluster 1 in the middle temporal gyrus (MTG) in Exp. 2 shows no significant group X imperceptibility interaction ($F(1,22)=0.59$, $p=0.45$). Error bars reflect mean squared error.

C. Data sampled from Cluster 2 in the superior temporal sulcus (STS) in Exp. 2 shows no significant group X imperceptibility interaction ($F(1,22)=1.4$, $p=0.25$). Error bars reflect mean squared error.

D. Data sampled from Cluster 3 in the inferior frontal gyrus (IFG) in Exp. 2 shows no significant group X imperceptibility interaction ($F(1,22)=0.65$, $p=0.43$). Error bars reflect mean squared error.

E. Data sampled from Cluster 4 in the ATL pole in Exp. 2 shows no significant group X imperceptibility interaction ($F(1,22)=0.18$, $p=0.68$). Error bars reflect mean squared error.

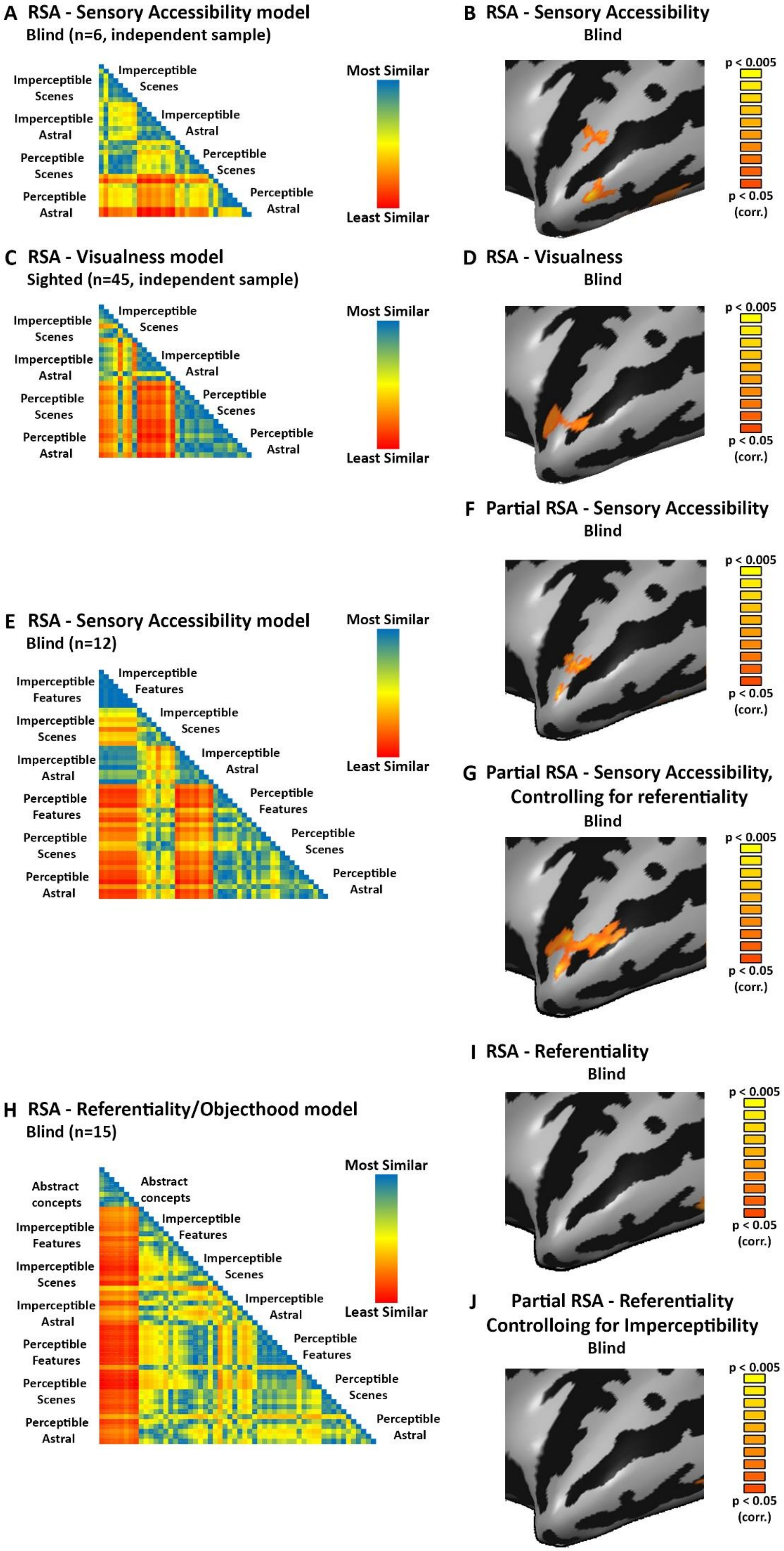
A RSA - Sensory Accessibility model
Blind (n=6, independent sample)
Imperceptible Scenes
Imperceptible Astral
Perceptible Scenes
Perceptible Astral
Most Similar
Least Similar
B RSA - Sensory Accessibility
Blind
p < 0.005
p < 0.05 (corr.)
C RSA - Visualness model
Sighted (n=45, independent sample)
Imperceptible Scenes
Imperceptible Astral
Perceptible Scenes
Perceptible Astral
Most Similar
Least Similar
D RSA - Visualness
Blind
p < 0.005
p < 0.05 (corr.)
F Partial RSA - Sensory Accessibility
Blind
p < 0.005
p < 0.05 (corr.)
E RSA - Sensory Accessibility model
Blind (n=12)
Imperceptible Features
Imperceptible Scenes
Imperceptible Astral
Perceptible Features
Perceptible Scenes
Perceptible Astral
Most Similar
Least Similar
G Partial RSA - Sensory Accessibility, Controlling for referentiality
Blind
p < 0.005
p < 0.05 (corr.)
I RSA - Referentiality
Blind
p < 0.005
p < 0.05 (corr.)
H RSA - Referentiality/Objecthood model
Blind (n=15)
Abstract concepts
Imperceptible Features
Imperceptible Scenes
Imperceptible Astral
Perceptible Features
Perceptible Scenes
Perceptible Astral
Most Similar
Least Similar
J Partial RSA - Referentiality Controlloing for Imperceptibility
Blind
p < 0.005
p < 0.05 (corr.)

Supplementary Figure 4: Neural pattern in dorsal ATL reflects imperceptibility in the blind

A-B. Multivariate representational similarity analysis (RSA) was computed based on a behavioral matrix based on ratings of the sensory perceptibility/accessibility of the astral and scene concepts collected from an independent group of congenitally blind subjects, who did not participate in the fMRI experiment (RDM in panel **A**, n=6). These ratings were highly correlated to those of the fMRI participants ($r^2=0.81$, $p<0.001$). Neural pattern correlation (**B**) to these ratings (data from Exp.2) was also found in the dorsal ATL.

C-D. RSA was computed also based on a behavioral matrix based on ratings of the "visualness" (visual dominance) of the astral and scene concepts collected from an independent group of sighted participants, who did not participate in the fMRI experiment (panel **C**, n=45). These ratings were negatively correlated to those of the sensory perceptibility of the blind participants ($r^2=0.45$, $p<0.001$). Neural pattern correlation (**D**) to these ratings was also found in the dorsal ATL of the blind (data from Exp.2).

E-G. RSA was computed also based on ratings of the blind subjects of the sensory perceptibility/accessibility of the concepts (panel **E**, replica of **Fig. 3A**), with the neural patterns in a searchlight manner across the brain while using behavioral ratings of abstractness, imaginability, manipulability, emotional valence and emotional arousal as nuisance regressors. Neural pattern correlation (**F**) was found in the dorsal ATL, overlapping the effects of imperceptibility X group interaction and abstract concepts preference, controlling for any collinearity between sensory perceptibility ratings and other factors (data from Exp.2). The same is found when also including referentiality as a nuisance regressor (panel **G**), indicating that the effect of imperceptibility is separable from this effect.

H-J. RSA was computed also based on ratings of a group of blind subjects of the referentiality of the concepts (n=15, panel **H**), with the neural patterns in a searchlight manner across the brain alone (panel **I**) or while using behavioral ratings of perceptibility, abstractness, imaginability, manipulability, emotional valence and emotional arousal as nuisance regressors (panel **J**). Referentiality/objecthood was defined as the extent to which each concept describes something that could be pointed out in the external world. Referentiality neural pattern correlation (**I,J**) was not found in ATL in either the blind group (presented here; data from Exp. 2) or sighted group. This may be due to an underpowered analysis, as only the abstract concepts show reduced referentiality as compared to the other concepts (data from Exp. 2).

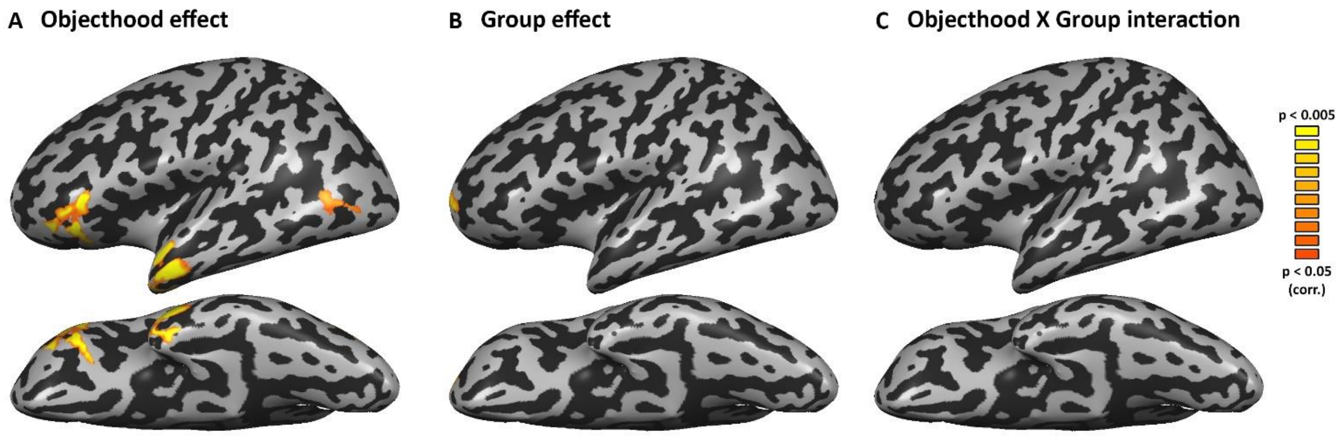

Supplementary Figure 5: Group effects in the comparison between abstract and imperceptible astral concepts (objecthood)

A 2-way ANOVA was computed for Objecthood (**A**; abstract vs. imperceptible astral concepts) and Group effects (**B**; blind, sighted; data from Exp.2). While the Objecthood effect is found in left ATL across groups, neither group effect or abstractness X group interaction (**C**) is found in this region.

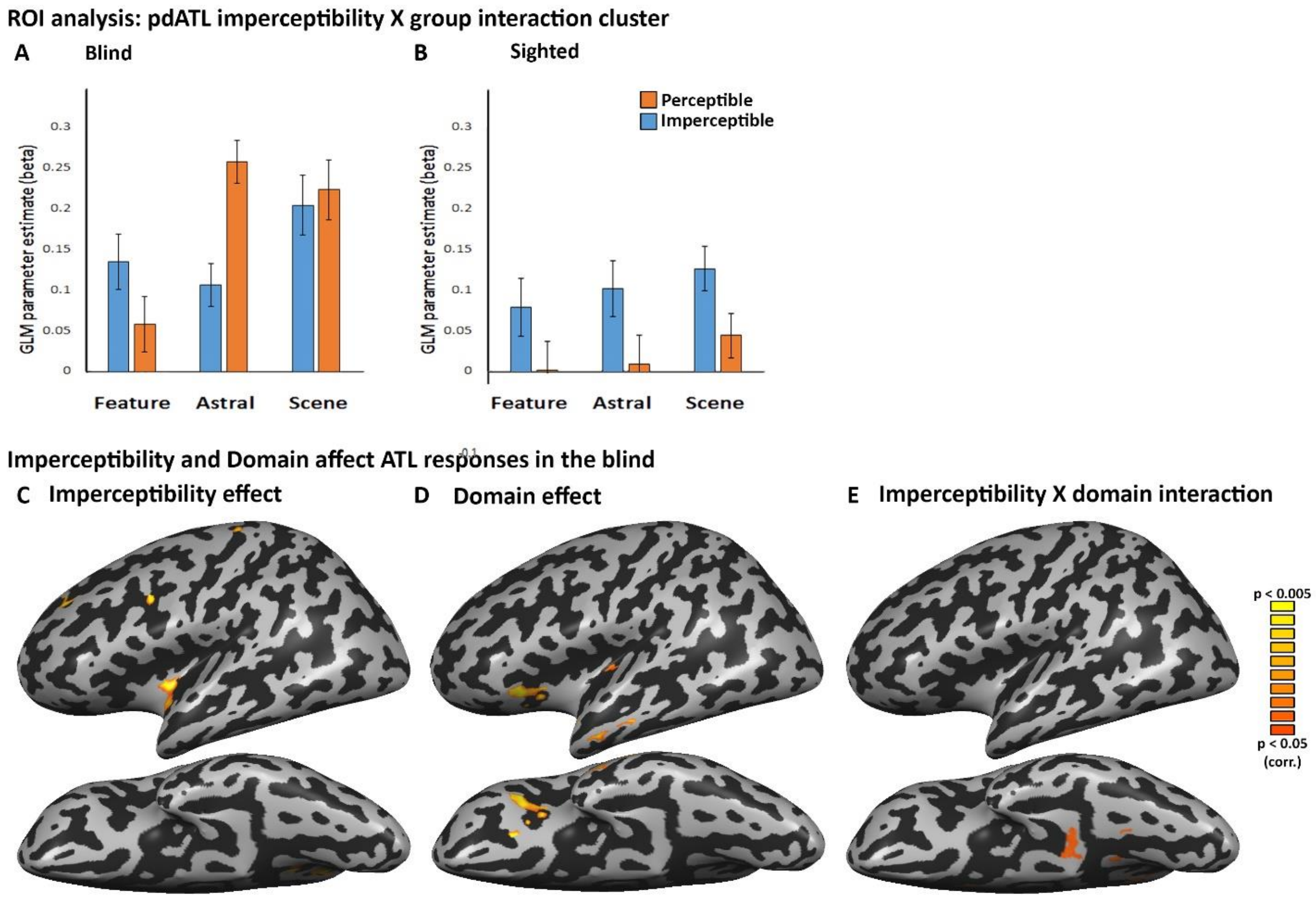

Supplementary Fig. 6: Imperceptibility and domain interaction

- **A.** Data sampled from the imperceptibility X group interaction cluster pdATL shown in **Fig. 2A** shows that this area does not show a consistent imperceptibility effect across content domains in the blind group ($F(2,11)=1.7$, $p<0.21$; data sampled from Exp. 2). Instead, it shows an interaction between imperceptibility and content domain in the blind ($F(2,22)= 5.73$, $p< 0.01$). Error bars represent standard error of the difference between means for the perceptible and imperceptible words in each content domain.
- **B.** Data sampled from pdATL in the sighted group (from Exp. 2). The interaction of imperceptibility with group, evident in pdATL in **Fig. 2A** appears to result from a bias in the sighted group, towards concepts which are imperceptible to the blind. This cannot be explained in terms of perceptibility, as all these concepts are similarly perceptible to the sighted. Error bars represent standard error of the difference between means for the perceptible and imperceptible words in each content domain.
- **C.** We computed a 2-way ANOVA for imperceptibility and content domain in the blind to inspect the interaction between them separately from the group effect (data from Exp.1). The imperceptibility effect in dorsal ATL replicates the findings of the contrast of imperceptible vs. perceptible concepts in the blind (**Fig. 2C**), showing that this effect is found regardless of content domain and depends on imperceptibility of the concepts only.
- **D.** Content domain effects in the blind are found in the inferior ATL as well as in the IFS (data from Exp.1).
- **E.** Whole-brain imperceptibility X domain interaction in the blind (data from Exp.1). Despite the significant interaction between imperceptibility and domain in the pdATL ROI, this effect does not survive a stricter whole-brain analysis. In ATL, interaction is found only in the uncus, in medial ATL. However, this region shows insignificant or negative BOLD in response to our experimental conditions

in Exp. 2, making the interaction problematic to interpret. Weaker interaction is found more posteriorly, in the parahippocampus, potentially due to the scene category[1,2] .

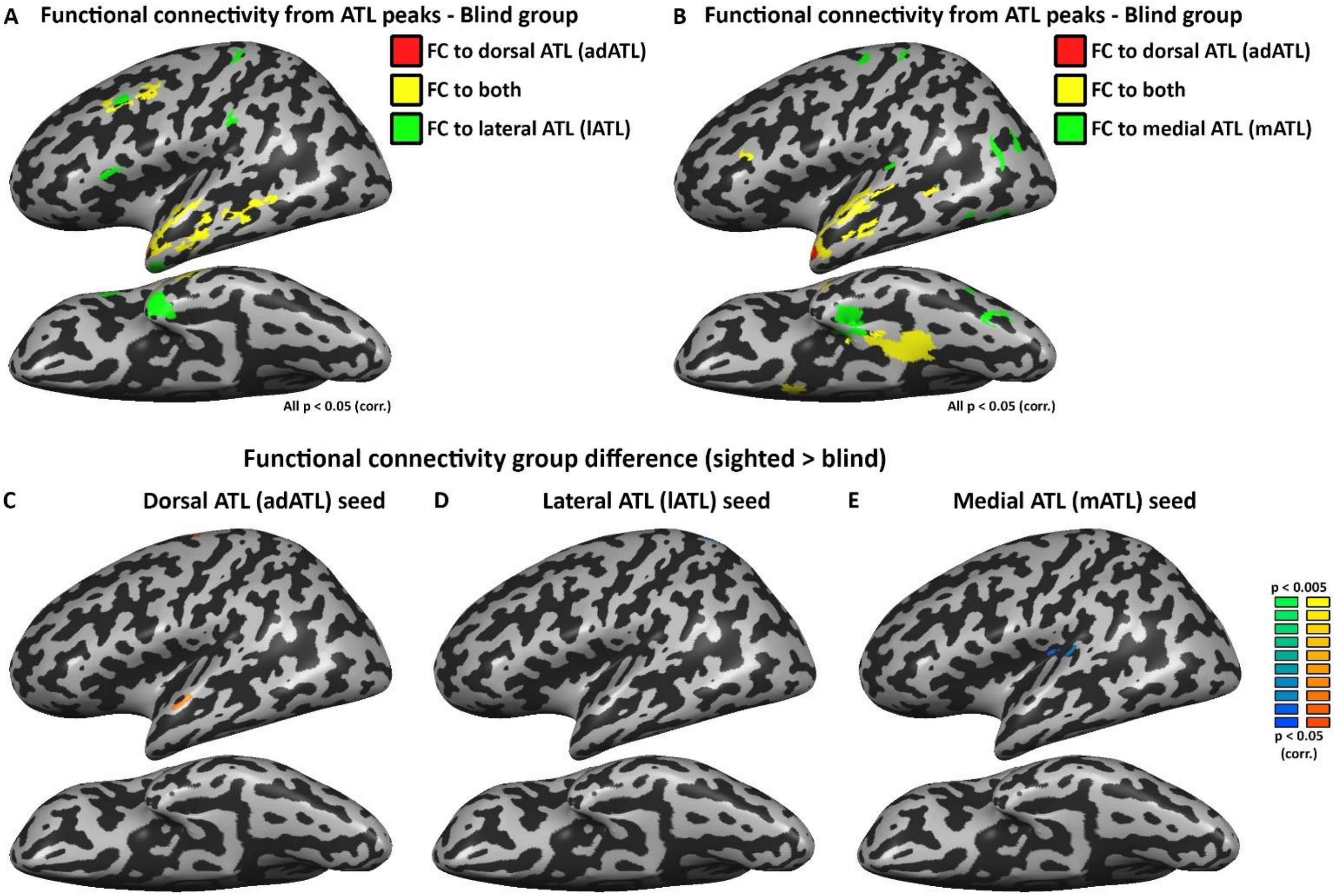

Supplementary Figure 7: Functional connectivity of ATL in the blind

Functional connectivity of the ATL peaks was computed from pairs of ATL seeds reflecting differential processing: the dorsal vs. lateral ATL (**A**; adATL and lATL) and dorsal vs. medial ATL (**B**; adATL and mATL). Although the dorsal and medial sites seem to share more connectivity in the blind (**B**) than in the sighted (**Fig. 6B**), no large-scale RSFC group effects are found for any of the ATL seeds (**C,D,E**).

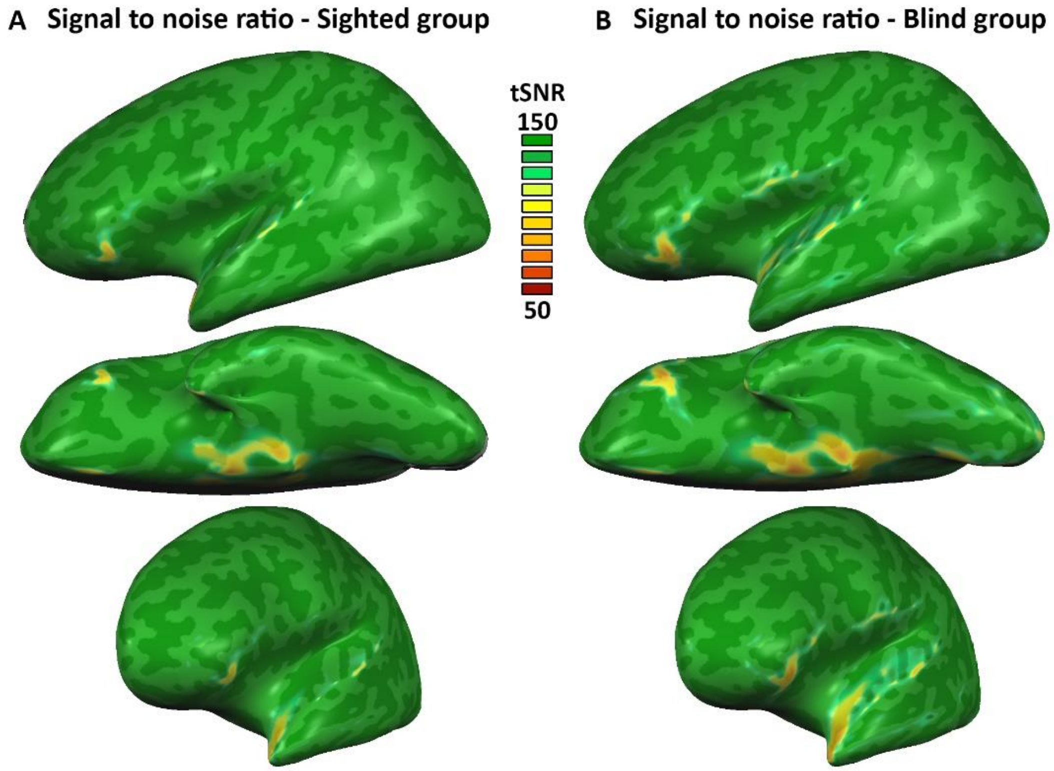

Supplementary Figure 8: Temporal signal to noise ratio in imaging ATL

the averaged temporal signal-to-noise ratio (tSNR, the ratio of the average signal intensity to the signal standard deviation) is presented for each group (**A**: sighted group, **B**: blind group). The maps show high quality signal coverage over ATL, although values are lowest in the ATL pole. It is possible that in the ATL pole relatively lower SNR could lead to low detection power, which may be improved using additional measures in future studies.

Supplementary Tables

Supplementary Table 1: Stimulus ratings (average across all 10 words in each category)

	Concreteness/ Abstractness	Familiarity	Age of acquisition	Imageability	Emotional arousal	Emotional valence	Semantic Diversity
**Objects**	6.50	6.25	4.29	6.75	5.2	3.2	1.46
**Imperceptible astral**	5.75	5.75	4.38	6.08	5.4	3.6	1.52
**Perceptible astral**	5.50	5.42	4.58	5.75	3.8	4.1	1.52
**Imperceptible scenes**	6.08	5.08	5.17	6.08	5.1	3.7	1.42
**Perceptible scenes**	6.00	5.50	4.63	6.00	4.8	3.6	1.62
**Imperceptible features (Colors)**	4.75	5.42	4.63	5.83	6.2	3.4	1.71
**Perceptible features (Shapes)**	5.50	5.83	4.63	6.08	6.4	4	1.62
**Abstract**	1.92	5.92	4.92	2.00	3.9	3.1	1.82

Supplementary Table 2: Stimulus differences (student's t-test p values)

Contrast	Concreteness/ Abstractness	Familiarity	Age of acquisition	Imageability	Emotional arousal	Emotional valence	Semantic Diversity
**Abstract vs. concrete**	0.000*	0.17	0.17	0.000*	0.06	0.84	0.003*
**Overall imperceptible vs. perceptible design[1]**	0.30	0.49	0.54	0.80	0.03	0.12	0.056
**Abstract vs. imperceptible astral**	0.000*	0.67	0.30	0.000*	0.03	0.27	0.03

* Statistically significant; p values are Bonferroni corrected for multiple comparisons.

[1] The overall imperceptible vs. perceptible design includes the comparison between the imperceptible and perceptible words in the three tested domains: astral/weather phenomena (e.g. "rainbow" vs. "rain"), scenes ("island" vs. "beach") and object features (colors vs. shapes, e.g. "red" vs. "square").
We first tested for the difference between the imperceptible and perceptible concepts using a mixed-effects ANOVA. The results show that there is no significant difference between the two concept types across measures ($F(1,58)=0.1$, $p=0.76$, $\eta^2= 0.0017$), but they did yield a trend for an interaction between concept type and behavioral measure ($F(3.1,177.7)=2.25$, $p = 0.08$, $\eta^2= 0.039$; As Mauchly's test indicated that the assumption of sphericity had been violated, $\chi^2(20)=212.5$, $p<0.001$, degrees of freedom were corrected using Greenhouse-Geisser estimates of sphericity, $\varepsilon=0.51$). Therefore, post-hoc Welch t-test contrasts were performed per behavioral measure, corrected for multiple comparisons.

Supplementary Table 3: Behavioral rating independent blind subjects group characteristics

Subject	Gender	Age	Years of Education*	Onset of Blindness	Cause of Blindness	Light Perception	Visual Memory
BS1	F	42	9	Birth	Congenital eyeball dysplasia due to teratogenic medication exposure	None	None
BS2	M	32	15	Birth	Congenital eyeball dysplasia	Faint	None
BS3	F	28	9	Birth	Congenital glaucoma	None	None
BS4	M	28	9	Birth	Congenital eyeball dysplasia	Faint	None
BS5	M	38	15	Birth	Congenital panophthalmitis due to inflection during pregnancy	None	None
BS6	F	31	15	1 year	Penicillin allergy	None	None
BS7	M	29	0	Birth	Congenital anophthalmia	None	None
BS8	M	39	12	Birth	Congenital microphthalmus/congenital eyeball dysplasia	None	None
BS9	M	33	9	Birth	Congenital optic atrophy	None	None

* Subjects BS1-BS6 rated the stimuli for perceptibility prior to the fMRI scan. No difference in education between behavioral ratings independent group and main fMRI group (Welch $t(6)=0.17$, $p<0.86$)

** Subjects BS7-BS9 along with the fMRI blind participants rated the stimuli for referentiality.

Supplementary References

1 He, C. *et al.* Selectivity for large nonmanipulable objects in scene-selective visual cortex does not require visual experience. *Neuroimage* **79**, 1-9, doi:http://dx.doi.org/10.1016/j.neuroimage.2013.04.051 (2013).

2 Wolbers, T., Klatzky, R. L., Loomis, J. M., Wutte, M. G. & Giudice, N. A. Modality-independent coding of spatial layout in the human brain. *Curr Biol* **21**, 984-989, doi:S0960-9822(11)00480-5 [pii]

10.1016/j.cub.2011.04.038 (2011).
